# Mapping Cell Atlases at the Single‐Cell Level

**DOI:** 10.1002/advs.202305449

**Published:** 2023-12-25

**Authors:** Fang Ye, Jingjing Wang, Jiaqi Li, Yuqing Mei, Guoji Guo

**Affiliations:** ^1^ Bone Marrow Transplantation Center of the First Affiliated Hospital, and Center for Stem Cell and Regenerative Medicine Zhejiang University School of Medicine Hangzhou Zhejiang 310000 China; ^2^ Liangzhu Laboratory Zhejiang University Hangzhou Zhejiang 311121 China; ^3^ Zhejiang Provincial Key Lab for Tissue Engineering and Regenerative Medicine Dr. Li Dak Sum & Yip Yio Chin Center for Stem Cell and Regenerative Medicine Hangzhou Zhejiang 310058 China; ^4^ Institute of Hematology Zhejiang University Hangzhou Zhejiang 310000 China

**Keywords:** cell atlas, integrative biology, single cell

## Abstract

Recent advancements in single‐cell technologies have led to rapid developments in the construction of cell atlases. These atlases have the potential to provide detailed information about every cell type in different organisms, enabling the characterization of cellular diversity at the single‐cell level. Global efforts in developing comprehensive cell atlases have profound implications for both basic research and clinical applications. This review provides a broad overview of the cellular diversity and dynamics across various biological systems. In addition, the incorporation of machine learning techniques into cell atlas analyses opens up exciting prospects for the field of integrative biology.

## Graphical abstract

The review discusses the technical characteristics of emerging single‐cell omics technologies and their diverse applications in various biological contexts, ranging from whole‐organism and single‐tissue analyses to studies involving evolution, development, aging, and diseases. It also provides insights into the incorporation of machine learning techniques into cell atlas analyses, providing exciting prospects for integrative biology.

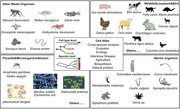

## 1 Introduction

Cells are the basic building blocks of animals, serving as both structural and functional units. They have different types and functions across tissues and organs, and their molecular networks play crucial roles in defining cell identities. Advances in global projects such as the Mouse Cell Atlas^[^ ^1^ ^]^ and Human Cell Atlas^[^ ^2^ ^]^ have greatly expanded our knowledge of cell biology by creating comprehensive whole‐organism cell atlases across various species. Whole‐organism cell atlases provide a powerful framework for systematically studying cell diversity, genetic networks, tissue organization, and disease processes. They offer an integrative view of biological systems, enabling researchers to observe cell type hierarchy from a global view. In addition, the construction of a high‐resolution immune‐cell atlas has facilitated a deeper understanding of the immune system and its diverse cell populations.^[^ ^3^ ^]^ Cell‐lineage atlases have also shed light on cell types that have previously been poorly characterized, such as stromal cells^[^ ^4^ ^]^ and endothelial cells,^[^ ^5^ ^]^ allowing researchers to explore their functions and interactions in the context of different tissues and organs.

In this review, we provide an overview of the advancements in constructing a single‐cell atlas in the past decade. We discuss the principle and technical characteristics of emerging single‐cell omics technologies. We place a strong emphasis on introducing the diverse applications of single‐cell atlases in various biological contexts, ranging from whole‐organism and single‐tissue analyses to studies involving evolution, development, aging, and diseases. Finally, we introduce the cutting‐edge application of sequence‐based deep learning models to the interpretation of cell atlases at the single‐cell level.

## 2 Technical Advances in Single‐Cell Atlas Mapping

**Figure 1:**
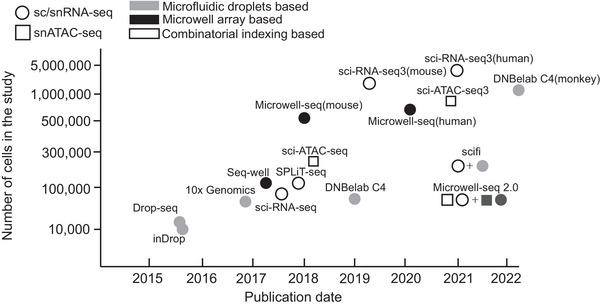
Representative single‐cell sequencing methods for cell atlas mapping. The approaches are denoted by two shapes as shown in the figure key, which refer to single‐cell or single‐nucleus transcriptome sequencing (sc/snRNA‐seq) and single‐nucleus assay for transposase accessible chromatin with high‐throughput sequencing (snATAC‐seq). Different colors represent the single‐cell library preparation strategy.

Since 2009, single‐cell RNA‐sequencing (scRNA‐seq) has become a powerful tool to study genomics at a single‐cell resolution.^[^ ^6^ ^]^ However, manual manipulation of single‐cell isolation limits the throughput and application of this technology in a variety of fields. Cell sorting methods (fluorescence‐activated cell sorting, [FACS]) have improved the throughput of single‐cell analysis to a certain extent. Soon after the establishment of scRNA‐seq protocols, multiwell plate‐based methods with high‐sensitivity were developed.^[^ ^7^ ^]^ However, the cost of library preparation and next‐generation sequencing (NGS) has hindered the mapping of large‐scale single‐cell landscapes on a higher order of magnitude. Only recently have massively parallel high‐throughput single‐cell sequencing platforms^[^ ^8^ ^]^ enabled profiling of cell atlases in many species (**Figure** **1**). Common cell atlas consortium databases usually contain more than 10 million single cells, while a complex organism is made up of trillions of cells. Therefore, an increase in sampling scale and multiomics will be important for comprehensive cell atlas construction in the future. A recent review summarized the latest advances in single‐cell multiomics technologies.^[^ ^9^ ^]^ Here, we will discuss different high‐throughput methods: transcriptomic methods, genomic methods, proteomic methods, and spatial methods for cell atlas mapping. These high‐throughput methodologies cover multidimensional information such as transcriptome, genome, and spatial distributions in various cell types.

### 2.1 Transcriptomic Methods for Cell Atlas Mapping

Gene expression is a critical dimension in defining the cellular state. High‐throughput scRNA‐seq methods involve three major strategies: droplets, microwells, and split‐pool barcoding. Representative droplet‐based scRNA‐seq methods include Drop‐seq^[^ ^8a^ ^]^ and inDrop.^[^ ^8b^ ^]^ In the droplets, the transcripts in every single cell are captured by oligo‐dT beads, followed by reverse transcription, DNA library amplification, and sequencing. Commercialized platforms such as 10× Genomics,^[^ ^10^ ^]^ M20 Genomics,^[^ ^11^ ^]^ and DNBelab C4^[^ ^12^ ^]^ that use the crossing channel droplet generation device have enabled massively parallel scRNA‐seq for large‐scale cell atlases. Further efforts in cell pre‐indexing have enabled an overload of cells from different samples in a single experiment.^[^ ^13^ ^]^ Thus, the throughput in one experiment could reach hundreds of thousands of single cells. Other commercialized microfluidic droplet platforms such as WaferGen ICELL8^[^ ^14^ ^]^ and Bio‐Rad ddSEQ Single‐Cell Isolator^[^ ^15^ ^]^ have limitations in throughput, which limits their application in cell atlas construction.

Microwell‐based high‐throughput single‐cell RNA‐seq methods utilize the microwell array to trap single cells and barcoded beads by gravity.^[^ ^16^ ^]^ Microwell‐seq^[^ ^1^ ^]^ and Seq‐well^[^ ^17^ ^]^ further optimize DNA amplification using the template‐switch strategy to capture the full‐length transcriptome. The advantages of the microwell array include the compatibility of different cell types and the ability to filter debris which may block the channels in droplet generation devices. Commercialized microwell array platforms such as BD Rhapsody^[^ ^18^ ^]^ and Singleron GEXSCOPE^[^ ^19^ ^]^ offer more options for standardized cell atlas mapping.

Initial efforts for multiplexed single‐cell combinatorial barcoding focused on copy number variation (CNV) profiling.^[^ ^20^ ^]^ Later, sci‐RNA‐seq^[^ ^21^ ^]^ and SPLiT‐seq^[^ ^22^ ^]^ demonstrated powerful instrument‐free characteristics for high‐throughput scRNA‐seq. The split‐pool strategy can generate millions of barcode combinations to profile the cell landscape in one experiment with indices to distinguish cells from multiple tissues or embryos.^[^ ^23^ ^]^ Combinatorial indexing methods provide another promising method of constructing a cell atlas in a cost‐effective manner.

### 2.2 Genomic Methods for Cell Atlas Mapping

Due to the cost of library preparation and sequencing, single‐cell whole genome sequencing (WGS) is rarely used in single‐cell atlas construction. However, high‐throughput single‐cell epigenomics sequencing has been applied to profile chromatin accessibility and methylation (e.g., snM‐seq) at a single‐cell resolution. In a single‐cell assay for transposase‐accessible chromatin by sequencing (scATAC‐seq), Tn5 transposase introduces a cell barcode during tagmentation. The inserted adapters are then ready to be captured by hybridization oligos in barcoded beads. Library preparation steps in scATAC‐seq are compatible with droplet‐ or microwell‐based platforms.^[^ ^24^ ^]^ A high‐throughput combinatorial indexing approach for scATAC‐seq was also developed to profile the chromatin accessibility of comprehensive cell types in a tissue‐level cell atlas.^[^ ^25^ ^]^ More importantly, multi‐omics methods for simultaneously profiling the transcriptome and chromatin accessibility enabled integrative analysis of gene expression regulation events in specific cell types.^[^ ^26^ ^]^ DNA methylation is another key epigenetic property that drives genetic regulation. Robust single‐cell DNA methylation methods based on multiwell plates^[^ ^27^ ^]^ and combinatorial indexing^[^ ^28^ ^]^ have greatly improved the throughput of DNA methylation sequencing to construct the DNA methylation atlas.^[^ ^29^ ^]^

### 2.3 Proteomic Methods for Cell Atlas Mapping

High‐throughput single‐cell proteome profiling approaches include sequencing‐based and mass spectrometry (MS)‐based methods. However, it remains challenging to detect the total proteins in a single cell or to directly sequence amino acids. Sequencing‐based methods such as CITE‐seq^[^ ^30^ ^]^ and REAP‐seq^[^ ^31^ ^]^ label cell membranes or nuclear proteins using oligo‐conjugated antibodies. The DNA barcodes in oligos are analyzed at the relative quantification level to describe the expression patterns of proteins. Alternatively, CyTOF,^[^ ^32^ ^]^ which combines metal ion‐modified antibodies and FACS, can be used to easily profile the target proteins in millions of cells. This method has been used to generate a protein atlas of immune responses in COVID‐19 infection.^[^ ^33^ ^]^

### 2.4 Spatial Methods for Cell Atlas Mapping

The spatial multimodal information of cell types in organs and embryos will facilitate the understanding of development and disease. Time‐course sampling combined with a 3D spatial atlas will help construct a complete single‐cell atlas in humans and other species.^[^ ^34^ ^]^ Both sequencing‐based and imaging‐based methodologies have been adopted for cell atlas construction. Sequencing‐based methods label the spatial location of RNA or DNA using barcoded probes that are printed on chips or beads. The DNA or RNA fragments are recovered and then sequenced on an NGS platform. Precoded coordinates are reconstructed from the sequencing data. NGS‐based spatial transcriptomics approaches emerged from “spatial transcriptomics” (ST)^[^ ^35^ ^]^ in 2016 and have been commercialized by 10× Genomics (Visium). The coordinate barcoded oligo‐dT probes are anchored on a glass slide to capture the poly‐A mRNA. The pixel size resolution of a spot is ≈50–100 µm, which means that the resolution has not reached the single‐cell level. In subsequent methods, the barcoded oligo‐dT primers are modified on beads (2–10 µm) to achieve single‐cell resolution. HDST^[^ ^36^ ^]^ and Slide‐seq^[^ ^37^ ^]^ utilize these barcoded beads to make monolayer bead distributions on silicon wafers or glass slides. The limitations of these methods are the tedious process of in situ sequencing and the imaging of the spatial barcodes on beads. To solve this problem, direct decoding of spatial barcodes using an NGS platform is the preferred option for other representative spatial transcriptomics approaches, such as Seq‐Scope,^[^ ^38^ ^]^ polony‐indexed library‐sequencing (Pixel‐seq),^[^ ^39^ ^]^ and spatial enhanced resolution omics sequencing (Stereo‐seq).^[^ ^40^ ^]^ Seq‐Scope uses the Illumina NGS sequencer to decode the barcode arrays as sequencing clusters. Stereo‐seq uses the MGI NGS sequencer and nanoball (DNB) sequencing chemistry to generate oligo‐dT‐modified DNB arrays to capture mRNA in tissue sections. This method has been used to construct high‐resolution single‐cell spatial transcriptomics atlases of mouse embryos and macaque cortex.^[^ ^41^ ^]^

Another technology roadmap for spatial barcoding is to deliver precoded primers through multiple microfluidic channels. The combination of horizontal and vertical coordinates defines a spatial barcode of a spot. The resolution of a single spot could range from 15 to 50 µm. Deterministic barcoding in tissue for spatial omics sequencing (DBiT‐seq)^[^ ^42^ ^]^ utilizes a strategy based on a multichannel microfluidic chip. Barcode ligation in the *X*‐ and *Y*‐axes of a specific grid is identified in sequencing data. Multiomics labeling of RNA and DNA using the microfluidic delivery system also is compatible with epigenomics profiling.^[^ ^43^ ^]^ All of these approaches are designed to capture and sequence RNA or DNA molecules in tissue sections without an accurate cell segmentation process. sci‐Space^[^ ^44^ ^]^ and slide‐tag^[^ ^45^ ^]^ combine previous high‐throughput single‐cell RNA‐seq with spatial barcoding. Decoded spatial oligos are permeated into fixed tissue sections. Then, labeled single nuclei in tissue sections are dissociated to proceed with scRNA‐seq, and the sequencing data of a real single cell are directly restored with the spatial distribution. More importantly, the sensitivity of high‐throughput scRNA‐seq of dissociated cells is better than that of in situ reverse transcription in fixed tissue sections.

Imaging‐based methods use a single‐molecule fluorescence in situ hybridization strategy to image transcripts at a subcellular level.^[^ ^46^ ^]^ Oligo probes of target transcripts detect gene expression with superresolution and high sensitivity. However, complex hybridization and imaging processes with limited target gene numbers are the main impediments to these methods. Multiplexed error‐robust FISH (MERFISH)^[^ ^47^ ^]^ and sequential FISH (seqFISH)^[^ ^48^ ^]^ greatly improve the throughput of transcript detection. Multicolor cycling imaging protocols enable the coding of hundreds to thousands of transcripts in one experiment. seqFISH+ takes advantage of superresolution imaging to detect more than ten thousand transcripts in a single cell.^[^ ^49^ ^]^ These methods have been used to profile the spatial cell atlases of the mouse primary motor cortex and mouse organogenesis.^[^ ^50^ ^]^ Alternatively, spatially resolved transcript amplicon readout mapping (STARmap)^[^ ^51^ ^]^ and fluorescent in situ sequencing (FISSEQ)^[^ ^52^ ^]^ introduce in situ sequencing (ISS) chemistry to sequence padlock probe barcodes after rolling cycling amplification (RCA). Commercialized ISS spatial transcriptomic solutions based on multiplex fluorescence in situ hybridization included MERSCOPE (Vizgen, Cambridge, MA, United States) and CosMx (NanoString, Seattle, WA, United States). MERSCOPE has been used to map the whole mouse brain atlas with more than 4 million cells.^[^ ^53^ ^]^ A representative ISS spatial transcriptome solution based on RNA padlock probe and RCA is Xenium^[^ ^54^ ^]^ (10× Genomics, Pleasanton, CA, United States). Moreover, the GeoMx system (NanoString, Seattle, WA, United States) provides a solution for in situ profiling of the whole transcriptome and 150 target proteins in formalin‐fixed paraffin‐embedded (FFPE) and fresh frozen (FF) tissue samples at cellular and subcellular resolutions. Together, these spatial sequencing platforms will accelerate the generation of spatial resources for cell atlas studies.

## 3 Overview of the Human Single‐Cell Atlas

### 3.1 Advances in the Human Cell Atlas

Technical advances in high‐throughput single‐cell genomics have revolutionized our ability to characterize the cell composition in complex human tissues. Morphological features and multi‐omics information define the function of specific cell types in normal and disease states. The international Human Cell Atlas (HCA) represents the global collaborative effort to comprehensively profile the cell reference map of human tissues for understanding both human health and diseases using cutting‐edge single‐cell genomic approaches.^[^ ^2^ ^]^ Launched in 2016, the first milestone of the HCA consortium community will be the detailed single‐cell transcriptome atlas of normal human organs. High‐quality datasets generated by different researchers will be integrated as an open resource. To date, scientists in the HCA consortium community have profiled more than 50 million cells from over 30 types of human organs.^[^ ^55^ ^]^ In 2019, the NIH Human Biomolecular Atlas Program (HuBMAP) launched another research community to map the human body at single‐cell resolution.^[^ ^56^ ^]^ Scientific communities in China are also eager to contribute to this research front. In 2020, Han et al. from Zhejiang University reported the world's first cross‐tissue single‐cell transcriptome landscape (over 700 000 cells in more than 50 tissues) of comprehensive human organs.^[^ ^57^ ^]^ The human cell landscape (HCL) included more than 100 major cell clusters and over 800 defined cell subtypes. The systematic comparison between adult and fetal landscapes revealed the transcriptomic stochasticity of progenitor cells in the early lineage stage. In the same year, He et al. reported another single‐cell transcriptional profiling study of 15 organs in a normal adult individual.^[^ ^58^ ^]^ In 2022, the *Tabula Sapiens* consortium constructed another multiple‐organ, single‐cell transcriptomic human atlas with 475 distinct cell types.^[^ ^59^ ^]^ In the same year, Eraslan et al. used single‐nucleus RNA sequencing to analyze frozen, banked samples from eight healthy human organs from 16 donors.^[^ ^60^ ^]^ This study demonstrated a framework for frozen tissues as part of the Genotype‐Tissue Expression (GTEx) project. At the chromatin accessibility level, Zhang et al. reported the first scATAC‐seq atlas of over 1 million nuclei in 222 distinct cell types across 30 adult human tissues.^[^ ^61^ ^]^ This study provided ≈1.2 million candidate *cis*‐regulatory elements (cCREs) and further identified the correlation of fetal and adult human cCREs with human traits and diseases.

The potential breakthrough between cell atlases and disease (common diseases, immune system diseases, rare diseases, and cancer) will pave the way for clinical diagnosis using single‐cell genomics. The severity of coronavirus disease 2019 (COVID‐19) has also raised considerable interest in the application of cell atlases in disease research.^[^ ^62^ ^]^ The immune cell transcriptome atlas as well as paired T‐cell receptors (TCRs) and B‐cell receptors (BCRs) were used to assemble a comprehensive immune cell type reference database across adult donors and pediatric and adult patients with COVID‐19 infection.^[^ ^3^, ^63^ ^]^ These two studies provided resources for healthy and infected blood data and explained the different clinical outcomes in children and adults.

**Figure 2:**
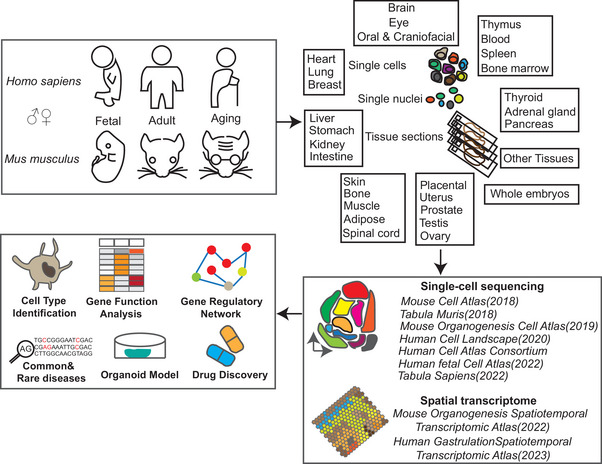
General procedures of human and mouse cell atlas construction. Currently, human (*Homo sapiens*) and mouse (Mus musculus) cell atlas have covered fetal (organogenesis and gastrulation), adult, and aging stage. For high‐throughput single‐cell/single‐nucleus sequencing, every individual tissues or organs are dissociated into single cells or single nuclei. For Spatial transcriptome sequencing, frozen or formalin‐fixed paraffin‐embedded (FFPE) tissue sections are obtained. Representative single‐cell atlas of human and mouse are listed in the box below. Function analysis and applications of cell atlas are shown in the left bow.

The Human Developmental Cell Atlas (HDCA) was launched as a part of the HCA. The aim of the HDCA is to create a comprehensive single‐cell reference map during normal organogenesis and early development stages to lay the foundation for aging, cancer, and regenerative medicine.^[^ ^64^ ^]^ The organoid cell atlas presents the first framework for drug development and therapies for cancer, rare genetic diseases, and multifactorial disorders.^[^ ^65^ ^]^ During the years 2017–2019, research teams from Qiao and Tang's laboratory reported a series of multiomics studies to map the development of human germline cells and embryo implantation.^[^ ^66^ ^]^ In 2020, the Genomic Architecture of Cells in Tissues (GeACT) project from their group and a another similar preprint multiomics study used high‐detectability scRNA‐seq and scATAC‐seq methods to profile the cell atlas of human fetuses during gestation (8‐21 weeks).^[^ ^67^ ^]^ Another two studies generated transcriptome and spatial datasets of the early gastrulating (16 and 19 days after fertilization) and organogenesis (4–6 weeks) stages of human embryos.^[^ ^68^ ^]^ Taking advantage of the modified version of sci‐RNA‐seq and sci‐ATAC‐seq, two papers presented the largest fetal human cell atlas (4 million single cells) from 28 fetuses ranging from 72 to 129 days in estimated postconceptual age.^[^ ^25^, ^69^ ^]^ In 2023, Pan and colleagues from BGI Genomics reported the first spatiotemporal transcriptome atlas of human embryos after gastrulation.^[^ ^70^ ^]^ They identified organ‐specific regulons as potential lineage‐determining factors. The cell type genes associated with developmental disorders and infection were classified. Combined with the adult human cell atlas, more resources for specific organ development are available (**Figure** **2**). Technological advances have contributed to the breadth (multiple tissues and stages) and precision (multiomics) of human cell atlas studies (**Table** **1**). A complete normal and disease cell atlas across different tissues will promote the accuracy of diagnosis and individualization of treatment.

**Table 1:** Advances in human tissue cell atlas.

Tissue	Methods	Type	Reference
Lung (Adult)	inDrop	RNA	[71]
Lung (Adult)	10×	RNA	[72a]
Lung (Adult)	10× & SmartSeq2	RNA	[72b]
Lung (Adult)	10× & Visium ST	RNA & VDJ	[73]
Lung (Integrated)	10× & SmartSeq2 & Seq well	RNA	[74]
Lung (Embryonic)	10× & Visium ST	RNA	[75a]
Lung (Embryonic)	10× & Visium ST	RNA & VDJ & ATAC	[75b]
Breast (Adult)	Fluidigm C1	RNA	[76a]
Breast (Adult)	10×	RNA	[76b]
Breast (Adult)	10×	RNA	[77]
Breast (Adult)	10×	RNA	[78a]
Breast (Adult)	10× & Visium ST	RNA	[78b]
Heart (Adult)	10×	RNA	[79]
Heart (Adult)	10× & Visium ST	RNA & ATAC	[80]
Heart (Embryonic)	10× & Visium ST & ISS	RNA	[81]
Intestine (Embryonic)	10×	RNA	[82a]
Gut (Multiple stages)	10× & SmartSeq2	RNA & VDJ	[82b]
Intestine (Embryonic)	10× & Visium ST	RNA	[83]
Intestine (Adult)	10× & CODEX	RNA & Protein & ATAC	[84]
Stomach (Adult)	10×	RNA	[85a]
Stomach (Adult)	CEL‐seq2	RNA	[85b]
Whole brain (Adult)	10×	RNA	[86]
Prefrontal cortex (Embryonic)	SmartSeq2	RNA	[87a]
Cerebral cortex (Embryonic)	STRT‐seq	RNA	[87b]
Cerebellum (Embryonic)	SPLiT‐seq	RNA	[87c]
Whole brain (Embryonic)	10×	RNA	[87d]
Hippocampus neurons (Multiple stages)	SPLiT‐seq	RNA	[88a]
Hippocampus glia (Multiple stages)	SPLiT‐seq	RNA	[88b]
Whole brain (Embryonic)	10× & Visium ST	RNA	[89]
Cornea (Adult)	10×	RNA	[90a]
Retina (Adult)	10×	RNA	[90b]
Kidney (Adult)	10× & SNARE‐seq2 & Visium ST & Slide‐seq2	RNA & ATAC	[91]
Kidney (Adult)	10× & CODEX & Mass spectrometry	RNA & Protein & Metabolomics	[92]
Kidney (Adult and Embryonic)	10×	RNA	[93]
Kidney (Adult)	10×	RNA & ATAC	[94]
Oral mucosa (Adult)	10×	RNA	[96]
Teeth (Adult)	10×	RNA	[97]
Liver (Adult)	CEL‐seq2	RNA	[99]
Liver (Embryonic)	10×	RNA	[100]
Pancreas (Adult)	CEL‐Seq2	RNA	[101a]
Pancreas (Adult and neonatal)	10× & ISS	RNA	[101b]
Pancreas (Embryonic)	MARS‐seq	RNA	[102]
Pancreas (Adult)	10×	RNA	[103]
Skin (Adult and Embryonic)	10×	RNA	[104]
Skin (Adult)	Mass spectrometry	Protein	[105]
Skin (Aging)	10×	RNA	[106]
Intervertebral disc (Adult)	10×	RNA	[107]
Spine (Embryonic)	10×	RNA	[108a]
Spine (Embryonic)	10× & Singleron	RNA & ATAC	[108b]
Limb (Adult and Embryonic)	10×	RNA	[109]
Skeletal muscle (Adult)	10×	RNA	[110a]
Skeletal muscle (Adult)	10×	RNA	[110b]
Limb (Embryonic)	10× & Visium ST	RNA	[111]
Prostate (Adult)	10×	RNA	[113]
Testis (Adult)	10×	RNA	[114]
Testis (Puberty)	10×	RNA	[115a]
Testis (Embryonic)	10×	RNA	[115b]
Testis (Aging)	10×	RNA	[116]
Uterus (Adult)	10×	RNA	[117]
Ovary (Adult)	10×	RNA	[118a]
Ovary (Adult)	10×	RNA	[118b]
Gonad (Embryonic)	10× & Visium ST	RNA & ATAC	[119]
Placenta	10× & Visium ST	RNA & ATAC	[120]
Placenta	10× & SmartSeq2	RNA	[121]
Placenta	MIBI‐TOF imaging & NanoString GeoMx	RNA & Protein	[122]
Whole body immune cells (Embryonic)	10× & Visium ST	RNA & VDJ	[123]
Whole body immune cells (Embryonic)	10× & SmartSeq2	RNA	[124]
Hemopoietic system (Embryonic)	10×	RNA	[125a]
Hemopoietic system (Embryonic)	10×	RNA	[125b]
Thymus (Multiple stages)	10×	RNA & VDJ	[126]
Spleen (Adult)	10×	RNA	[127]
Bone marrow (Embryonic)	10× & CITE‐seq	RNA & Protein	[128]
Blood (Adult)	STRT‐seq	RNA	[129]
Blood (Aging)	10×	RNA	[130b]

VDJ, V(D)J recombination; CODEX, co‐detection by indexing.^[^ ^131^ ^]^

### 3.2 Human Lung Cell Atlas

An early study of human and mouse lung proximal airway epithelial cells defined a new CFTR‐rich pulmonary ionocyte.^[^ ^71^ ^]^ Another study described the gene expression of multiple epithelial cells such as ionocytes, pulmonary neuroendocrine cells, and brush cells, from the nasal epithelium to successive divisions of the airways.^[^ ^72^ ^]^ Multiomics (spatial transcriptomics, snRNA‐seq, scRNA‐seq, TCR, BCR) sequencing from five locations (trachea, bronchi, and upper and lower parenchyma) across the human lung described the gland‐associated immune niche of B cells and plasma cells.^[^ ^73^ ^]^ Recently, as a part of the HCA, an integrated human lung cell atlas (HLCA) presented standard annotations of known, rare, and previously undescribed cell types.^[^ ^74^ ^]^ This reference database also identified shared cell states across multiple lung diseases including COVID‐19 and lung carcinoma. Another two independent studies mapped a multiomics cell atlas of lung samples from embryos at 5–22 weeks postconception.^[^ ^75^ ^]^

### 3.3 Human Breast Cell Atlas

Human breast single‐cell studies of mammary epithelial cells greatly improve the understanding of adult mammary glands and breast cancer.^[^ ^76^ ^]^ Analysis of human milk or nonlactating breast tissues revealed the secretory cell subtypes in milk, which offers a reference map between pregnancy, lactation, and breast cancer.^[^ ^77^ ^]^ Most recently, the Human Breast Cell Atlas (HBCA) consortium reported two large‐scale single‐cell and spatial atlases of parenchymal and immune cells. These studies illustrated the tissue‐resident immune cell ecosystem and evaluated the potential immune exhaustion events in noncancerous tissues during the very early stages of tumor initiation. The 1.5 million single‐cell resource data of human breasts represent another milestone in HCA.^[^ ^78^ ^]^

### 3.4 Human Heart Cell Atlas

Cardiovascular disease has the highest death rate among all diseases. Cardiomyocytes, stromal cells, and vascular endothelial cells in different heart regions are heterogeneous. Sarah Teichmann's group presented the most comprehensive single‐cell transcriptome atlas of six distinct cardiac regions in healthy adult hearts. The system comparison between cardiac cells and skeletal muscle cells highlights cardiac‐specific cell signatures.^[^ ^79^ ^]^ Most recently, they offered a multiomics and spatial atlas of adult human cardiac niches and introduced a drug‐ target prediction framework to decipher the chronotropic effects of drugs.^[^ ^80^ ^]^ In addition, the spatial annotation of human embryonic cardiac cell types at 4.5–5, 6.5, and 9 weeks postconception was achieved using spatial transcriptome sequencing, in situ sequencing (ISS), and scRNA‐seq.^[^ ^81^ ^]^

### 3.5 Human Gut Cell Atlas

Research efforts in human fetal gut development have characterized intestinal morphogenesis and complicated cell types over time. Elmentaite and colleagues generated in‐depth single‐cell maps of the fetal and adult human intestine to compare cell type‐specific expression across space and time. They also predicted the gene‐associated programs in Hirschsprung's disease, inflammatory bowel disease (IBD), and pediatric Crohn's disease to reveal disease‐associated transcriptional changes.^[^ ^82^ ^]^ Alison Simmons's group used scRNA‐seq and spatial transcriptomic analysis to create a spatiotemporal atlas of human intestinal development from 8 to 22 weeks postconception. They described location‐specific cellular compartments and fate decision events of fibroblasts, vascular niche cells, and immune cells.^[^ ^83^ ^]^ Recently, advances in the Human BioMolecular Atlas Program (HuBMAP) integrated scRNA‐seq, scATAC‐seq, and protein multiplexed imaging approaches to construct a cell atlas of the small intestine and colon in healthy humans.^[^ ^84^ ^]^ Two other studies reported large datasets of the human upper gastrointestinal region (stomach) and identified stem cell signatures of basal cells and fibroblasts.^[^ ^85^ ^]^

### 3.6 Human Cell Atlas in the Central Nervous System

The largest adult HCA human brain cell atlas was sampled from over 3 million nuclei from ≈100 dissections across the forebrain, midbrain, and hindbrain. The unique cellular composition in anatomical locations and area‐specific neurons and glia was profiled.^[^ ^86^ ^]^ During 2018–2021, researchers also mapped the single‐cell landscapes of the prefrontal cortex and cerebellum in developing human embryos.^[^ ^87^ ^]^

In 2022, Song and Ming's laboratory constructed a single‐nucleus transcriptome atlas of immature dentate granule cells (imGCs) and glia in the human hippocampus at different stages across the lifespan.^[^ ^88^ ^]^ Recently, as a part of the HCA, Braun et al. combined scRNA‐seq and spatial transcriptomics to reveal cell states and trajectories in human brains at 5–14 weeks postconception. Specifically, they found abundant region‐specific glioblasts maturing into distinct preastrocytes and preoligodendrocyte precursor cells in the forebrain.^[^ ^89^ ^]^

The retina is a specialized neural tissue in the eye. Some efforts have also been made to profile the single‐cell transcriptome atlas of adult, embryonic, and fetal corneas from 10 to 21 weeks postconception.^[^ ^90^ ^]^ The human retina data in the adult and developmental stages will serve as a benchmarking resource of corneal organoids for studying and treating retinal disease.

### 3.7 Human Kidney Cell Atlas

The HuBMAP project reported a scRNA‐seq and spatial imaging dataset of approximately half a million cells in healthy reference and diseased kidneys.^[^ ^91^ ^]^ They defined the injury‐related active immune responses underlying epithelial repair. Another reference kidney atlas generated by the Kidney Precision Medicine Project (KPMP) also built high‐resolution transcriptomics, proteomics (CODEX imaging), and spatial metabolomics atlases of nephrectomy and normal tissues.^[^ ^92^ ^]^ To resolve the immune topology of the human kidney, Stewart et al. performed scRNA‐seq of nephron resident myeloid and lymphoid cells as well as other parenchymal cells.^[^ ^93^ ^]^ They analyzed the cross‐talk network between myeloid cells and epithelial cells in the regions that are most susceptible to infection. At the chromatin accessibility level, a scATAC‐seq dataset of adult human kidneys identified cell type‐specific regulons and transcription factor binding motifs in different proximal tubule epithelial cell subtypes.^[^ ^94^ ^]^

### 3.8 Human Oral and Craniofacial Cell Atlas

Human oral and craniofacial tissues are associated with multiple physiological processes and are composed of various cell lineages. A systematic review has summarized the roadmap and advances in oral and craniofacial tissues, such as the gingival mucosa, buccal mucosa, salivary glands, periodontium, and tonsils.^[^ ^95^ ^]^ Here, we introduce two efforts regarding the human oral mucosa and teeth. The human oral mucosa cell atlas compiled a transcriptome resource for healthy individuals and patients with periodontitis.^[^ ^96^ ^]^ They discovered the diversity of stromal and epithelial cells at the oral barrier. More importantly, they highlighted the role of fibroblasts in promoting the recruitment of neutrophils and expressing chemokines to recruit lymphocytes. The human teeth atlas revealed various cell populations in dental pulp and periodontium.^[^ ^97^ ^]^ The characterization of stem cell populations in different tooth compartments will inspire stem cell‐based dental therapeutic approaches.

### 3.9 Human Liver and Pancreas Cell Atlas

A recent review summarized single‐cell and spatial transcriptomics advances in liver biology, including liver homeostasis, development, regeneration, chronic liver disease, and cancer.^[^ ^98^ ^]^ In 2019, Aizarani et al. presented a high‐sensitivity adult human liver cell landscape to analyze heterogeneous epithelial populations.^[^ ^99^ ^]^ The human liver developmental atlas further represented the hepatocyte maturation process from the endoderm (2.5 weeks postconception) to 10 weeks postconception.^[^ ^100^ ^]^ They described the origin and differentiation process of cholangiocytes, hepatic stellate cells, and tissue‐resident Kupffer cells.

A high‐sensitivity human pancreatic atlas from adult and neonatal human donors has been reported.^[^ ^101^ ^]^ In 2021, Gonçalves et al. published a single‐cell transcriptome dataset spanning fetal development from 7 to 10 weeks postconception. They developed a system to allow for long‐term culture of human pancreatic progenitors.^[^ ^102^ ^]^ In 2022, Fasolino et al. adopted single‐cell transcriptome and proteomics approaches to map the pancreatic islet cell atlas from healthy, type 1 diabetes (T1D) and beta cell autoantibody‐positive donors. They found a similarity between MHC class II‐expressing ductal cells and dendritic cells in T1D patients.^[^ ^103^ ^]^

### 3.10 Human Skin Cell Atlas

The human skin cell atlas focused on the role of innate immune cells in clonal expansion of disease‐associated lymphocytes. The study identified vascular endothelial cell and macrophage clusters that interact with lymphocytes in atopic dermatitis and psoriasis.^[^ ^104^ ^]^ A spatially resolved quantitative proteomic atlas of healthy human skin identified undescribed proteins associated with immune activation functions in cellular subsets.^[^ ^105^ ^]^ In 2021, the human skin aging atlas characterized canonical and basal cell types in human eyelid skin from healthy individuals across different ages, which will inspire therapeutic strategies for aging‐related skin disorders.^[^ ^106^ ^]^

### 3.11 Human Bone and Muscle Cell Atlas

Gan et al. mapped the single‐cell transcriptomic landscape of human intervertebral discs. They classified the chondrocyte subtypes and resident progenitors with trilineage differentiation capacity.^[^ ^107^ ^]^ Two studies further revolved around a single‐cell transcriptome atlas in the entire human spine between 7 and 23 gestational weeks.^[^ ^108^ ^]^ They found stem cell characteristics in fibroblasts and neuroendocrine cells. Human embryonic long bones and limb buds were dissected at the single‐cell level to define the perichondrial embryonic skeletal stem/progenitor cell, which could contribute to osteochondral lineage cells.^[^ ^109^ ^]^

In 2020, two studies developed a human skeletal muscle cell atlas to identify muscle mononuclear cell types and muscle stem/progenitor cells.^[^ ^110^ ^]^ In 2023, HCA released its effort in the human embryonic limb cell atlas.^[^ ^111^ ^]^ They constructed a scRNA‐seq and spatial transcriptomic atlas of developing limbs between 5 and 9 weeks postconception and identified 55 subtypes. Musulin was identified as a key transcriptional repressor that maintains muscle stem cell identity. A cross‐species developmental comparison between human and mouse limbs identified homology in cell type and gene expression patterns.

### 3.12 Human Urinary and Reproductive System Cell Atlas

Cross‐species studies in humans and mice have revealed homology and heterogeneity in bladder epithelial cells.^[^ ^112^ ^]^ Functional epithelial subsets associated with nerve conduction and allergic reactions offer an in‐depth resource of the normal bladder. A single‐cell transcriptome atlas of normal human prostate tissue from different anatomical regions identified previously unrecognized epithelial cell types. These data will serve as a cellular baseline for understanding changes in human prostate disease.^[^ ^113^ ^]^

In 2018, the representative adult human testis transcriptional cell atlas identified a novel spermatogonial stem cell state with high developmental plasticity.^[^ ^114^ ^]^ Guo et al. reported a complete dynamic transcriptional cell atlas of the human testis at the embryonic, fetal, infant, and juvenile stages. They described the differentiation of Sertoli and interstitial cells at 6–7 weeks post‐fertilization. During puberty, they found two distinct transcriptional and metabolic states of Sertoli cells. A common prepubertal progenitor for Leydig and myoid cells was annotated to enrich the regulons controlling pubertal differentiation.^[^ ^115^ ^]^ In 2022, Guo's group further profiled the single‐cell transcriptome atlas of both young and aging individuals to examine age‐related changes in germline development and in testicular somatic cells. They found widely existing inflammation signaling perturbations in Sertoli cells, Leydig cells, and testicular peritubular cells.^[^ ^116^ ^]^

The HCA project further profiled the human uterus cell atlas and dissected the epithelial lineages in the luminal and glandular microenvironments.^[^ ^117^ ^]^ Two works provided a molecular map of the human adult ovary to distinguish granulosa and theca cells and other cell types.^[^ ^118^ ^]^ They validated the existence of germline stem cells in adult human ovaries.

A multi‐omics dataset of half a million cells in both human‐ and mouse‐defined gonadal somatic cells during early sex determination. They annotated a supporting‐like PAX8+ population to mediate the formation of the rete testis and rete ovary.^[^ ^119^ ^]^ The HCA and HuBMAP projects constructed high‐resolution transcriptome and spatial maps of the human placenta. Both studies highlighted the cell–cell communication events in the decidua and arterial transformation during early pregnancy.^[^ ^120^ ^]^ The regulation of immune responses between NKs and other cells during placentation was also explored using ligand–receptor interaction analysis.^[^ ^121^ ^]^ The latest spatial cell atlas utilized spatial proteomics and transcriptomics to study the human maternal–fetal interface in the first half of pregnancy. They revealed the immune tolerance‐conducive composition in the decidua.^[^ ^122^ ^]^

### 3.13 Human Immune and Hematopoietic System Cell Atlas

The HCA project constructed a multiomics cross‐tissue single‐cell atlas of developing human immune cells across prenatal hematopoietic, lymphoid, and nonlymphoid peripheral organs.^[^ ^123^ ^]^ Over 100 cell states in ≈1 million single cells revealed conserved gene signatures in the maturation process of monocytes and T cells. Another HCA study portrayed the human repertoire of human blood and immune cells during development. They demonstrated a shift in the hemopoietic composition of the fetal liver during gestation and differentiation trajectories of HSCs.^[^ ^124^ ^]^ To further define the ontogeny of human hematopoietic stem cells (HSCs), Calvanese et al. performed scRNA‐seq of hematovascular cells in the aorta–gonad–mesonephros (AGM) region of human embryos at 4.5–5 weeks. They documented the development and stage‐specific transitions of HSCs from arterial hemogenic endothelium to other tissues.^[^ ^125^ ^]^ Other single‐cell atlases of immune and hematopoietic system tissues including thymus,^[^ ^126^ ^]^ spleen,^[^ ^127^ ^]^ bone marrow,^[^ ^128^ ^]^ and blood,^[^ ^129^ ^]^ have provided abundant resources to study the development of the human immune system. The peripheral blood atlas of old men and supercentenarians evaluated aging‐associated hallmarks of frailty‐specific immune cell characteristics. An “aging clock” was established to evaluate the balance between ribosomal activity and inflammation.^[^ ^130^ ^]^

## 4 Overview of the Mouse Single‐Cell Atlas

### 4.1 Cross‐Tissue Cell Atlas of Adult Mice

As the most traditional model organism, *Mus musculus* has made great contributions to current biology and medicine. The cell atlas in normal mouse tissues serves as a reference cell map to decipher the perturbation of treatment in the cell state. Although cell atlases of some mouse tissues have been reported on a limited scale,^[^ ^132^ ^]^ the comprehensive cell type composition in mouse tissues still remains to be defined. Soon after the application of the high‐throughput scRNA‐seq method, in 2018, Han et al. reported the world's first groundbreaking mouse cell atlas (MCA), which includes comprehensive single‐cell transcriptome (nearly half a million cells) information in more than 40 organs and tissues.^[^ ^1^ ^]^ The MCA database represents an important resource for cell type identification in major organs and many previously unstudied tissues. Subsequently, the *Tabula Muris* Consortium reported another single‐cell transcriptome atlas of 20 mouse organs.^[^ ^133^ ^]^ They applied both full‐length and 3′‐end methods to profile the transcriptome and performed transcription factor analysis across multiple cell lineages. Additionally, in 2018, Cusanovich et al. reported the first single‐cell genome‐wide chromatin accessibility atlas comprising 13 adult mouse tissues. The cell type‐specific regulatory elements offered a framework to identify cell types principally related to common human diseases and traits.^[^ ^25a^ ^]^ Additionally, to delve into the functions and interactions of the same cell lineage in diverse tissues or regions, Zeisel et al. developed the first single‐cell transcriptome atlas of the mammalian nervous system. They dissected different anatomical regions of the adult brain and spinal cord, as well as the peripheral sensory, enteric, and sympathetic nervous systems.^[^ ^134^ ^]^ In 2020, Kalucka et al. constructed the first endothelial cell atlas from 11 mouse tissues and identified 78 subclusters.^[^ ^5^ ^]^ They described heterogeneous metabolic gene signatures in endothelial cells derived from different vascular beds. Collectively, these cell landscapes have produced valuable resources for discovering adult tissue cell types and facilitating cross‐species analysis (Figure 2).

### 4.2 Cross‐Tissue Cell Atlas of Developing and Aged Mice

Gastrulation and organogenesis are the two most important time points during embryo development. Mouse embryos provide a homologous model to discover these development processes. During mouse embryo gastrulation, lineage‐specific progenitors emerge, and most development‐associated genes in this stage have been studied.^[^ ^135^ ^]^ In 2019, two back‐to‐back studies represented large‐scale single‐cell transcriptome atlas of mouse gastrulation and organogenesis. Pijuan‐Sala et al. studied nine sequential time points ranging from 6.5 to 8.5 days post‐fertilization.^[^ ^136^ ^]^ In a parallel effort, Cao et al. profiled a transcriptome atlas of ≈2 million cells derived from 61 embryos staged between 9.5 and 13.5 days of gestation.^[^ ^23^ ^]^ During early endoderm development, major embryonic lineage analysis demonstrated gene expression transition and diversification. In contrast, the mouse organogenesis cell atlas provided a global view of the developmental processes of major cell lineages at a later stage. Furthermore, Peng et al. and others constructed spatial transcriptomic landscapes, highlighting specific cell fates during early gastrulation (embryonic day 5.5 (E5.5) and E7.5).^[^ ^137^ ^]^ Single‐cell multiomic methods have been widely used in embryo research. The first comprehensive histone modification and scATAC‐seq atlas of prenatal (E14.5), postnatal, and primitive gut tubes identified dynamic enhancers and key transcriptional regulators beyond gene expression.^[^ ^138^ ^]^ A companion single‐cell atlas from two modalities (RNA and ATAC) in E7.5 and E13.5 samples showed spatial patterns of *cis*‐elements and the spatial distribution of potentially functional transcription factors.^[^ ^139^ ^]^ The study also identified the existence of Sertoli and granulosa cell progenitors in gonads from both sexes.

In recent years, with the development of spatial methods and computational frameworks, more researchers have aimed to construct larger‐scale developmental state manifold landscapes of mouse embryos. For temporal profiling, Fei et al. reported a time‐series mouse cell differentiation atlas ranging from the early embryonic stage to the mature adult stage (E10.5 to postnatal day 21).^[^ ^140^ ^]^ They identified and validated Xbp1 as a lineage‐common master regulator in fate‐decision circuits in mice. Mittnenzweig and colleagues introduced another single‐embryo, single‐cell‐level transcriptome dataset of mouse gastrulation (E6.5 to E8.1). They built the most comprehensive coordinated dynamic cell lineage differentiation model to infer the differentiation flows.^[^ ^141^ ^]^ Qiu and colleagues constructed the largest scRNA‐seq dataset of mouse embryos thus far, encompassing gastrulation and postnatal stage, with an atlas of 12.4 million cells. The whole embryo snapshots curate a rooted tree of cell‐type relationships that spans mouse development from zygote to pup. A notable cell‐type transcriptional state shift from the placenta to extrauterine life was captured, which separated from their fetal progenitors.^[^ ^142^ ^]^ For spatial profiling, one spatial transcriptomic atlas used Slide‐seq to profile the whole embryo from E8.5 to E9.5 at the onset of organogenesis.^[^ ^143^ ^]^ This approach revealed distinct spatial patterns in neural tube mapping, uncovering previously unannotated genes. BGI developed the spatial transcriptomic method Stereo‐seq with subcellular resolution. They initiated the mouse organogenesis spatiotemporal transcriptomic atlas (MOSTA) to map the spatiotemporal transcriptomic dynamics during the development of mouse embryos from E9.5 to E16.5.^[^ ^144^ ^]^ This valuable resource presents the first full‐scale transcriptome dataset of mouse embryos during gastrulation and organogenesis, and will help more scientists discover transcriptomic dynamics at these critical stages.

The adult mouse cross‐tissue aging single‐cell transcriptome atlas, known as “*Tabula Muris Senis*,” was published in 2020. It highlighted the aging hallmarks such as mutational burden, genomic instability, and immune system gene expression phenotypes reflected aging‐related perturbations in a broad range of tissues.^[^ ^145^ ^]^ The cross‐species cell landscape also included the time‐related signatures throughout the life cycle of mouse aging.^[^ ^146^ ^]^ Notably, structural inflammation and mitochondrial dysfunction were identified as the most common hallmarks of organism aging. Cross‐species data serve as an invaluable resource to compare organism cell types at the single‐cell level (https://bis.zju.edu.cn/cellatlas/).

### 4.3 Mouse Cell Atlas in Central Nervous System

The mouse brain is the most well‐studied tissue among all organs. Recently, the BRAIN Initiative Cell Census Network (BICCN) took advantage of single‐cell genomics methods to construct a series of cellular databases in the mouse primary motor cortex.^[^ ^147^ ^]^ As the first milestone, BICCN generated the multimodal cell census and atlas of the mammalian primary motor cortex.^[^ ^148^ ^]^ The representative single‐cell atlas in these efforts includes representative single‐cell multiomics datasets (RNA‐seq and ATAC‐seq) of the mouse primary motor cortex. Yao and colleagues discovered thousands of concordant marker genes and gene regulatory elements for these cell types.^[^ ^149^ ^]^ Another atlas of gene regulatory elements in the adult mouse cerebrum linked cis‐regulatory elements to putative target genes expressed across diverse cerebral cell types.^[^ ^150^ ^]^ Furthermore, a single‐cell DNA methylation atlas from 45 regions of the mouse cortex, hippocampus, striatum, pallidum, and olfactory areas identified epigenetic signatures for more than 150 cell types. These insights were employed to construct an artificial neural network model to predict neuron identity, integrating regulatory elements, transcription factors, and spatial locations.^[^ ^29^ ^]^

A more recent spatial transcriptomic atlas of mouse brains demonstrated the power of high‐resolution approaches to map the whole brain architecture. Macosko and Chen's laboratory combined high‐throughput snRNA‐seq with Slide‐seq to establish a transcriptomics atlas of the entire mouse brain.^[^ ^151^ ^]^ This dataset enabled comprehensive characterization of neuropeptide and neurotransmitter signaling in each region‐specific cell type. Additionally, Zhuang's laboratory generated a high‐resolution cell atlas of brain aging within the frontal cortex and striatum using a spatially resolved in situ sequencing platform. More pronounced perturbation of spatial gene expression was observed in nonneuronal cells.^[^ ^152^ ^]^ In other central nervous system tissues, a single‐cell atlas of immune, glial, and retinal pigment epithelial cells from adult mouse retina revealed the temporal inflammatory patterns of infiltrating immune cells after optic nerve crush injury.^[^ ^153^ ^]^

### 4.4 Representative Mouse Cell Atlas of Other Tissues

In the mouse immune system, two representative works applied scRNA‐seq to trace and reconstruct the differentiation of mouse hematopoietic stem and progenitor cells.^[^ ^154^ ^]^ In 2019, the single‐cell atlas of the mouse bone marrow microenvironment revealed cellular heterogeneity within the bone marrow niche, revealing that chemotherapy‐induced perturbation resulted in downregulation of vascular Dll4 and a myeloid skewing event.^[^ ^155^ ^]^

Heart and lung development are critical events in mouse respiratory system. The single‐cell transcriptome atlas of mouse heart development identified gene expression modules enriched at the early embryonic and neonatal stages.^[^ ^156^ ^]^ This dataset indicated that mutants of the epicardium‐expressed genes Wt1 and Tbx18 result in different heart defects. On the other hand, the single‐cell atlas of mouse lung development and aging uncovered increased cholesterol biosynthesis in type 2 pneumocytes and lipofibroblasts as a hallmark of lung aging. Proteomic validation further revealed the perturbation of different types of collagens in aged mice.^[^ ^157^ ^]^

In the digestive system, numerous scRNA‐seq studies have profiled the heterogeneity of hepatocytes and fibroblasts in normal and treated mice.^[^ ^158^ ^]^ Moreover, the single‐cell landscapes of the epithelium in the small intestine, large intestine, and colon identified diverse enterocytes and secretory cells to maintain intestinal homeostasis.^[^ ^132^, ^159^ ^]^ In the murine pancreatic development cell atlas, researchers identified previously undescribed endocrine progenitors and candidate transcriptional regulators for alpha or beta cell lineages.^[^ ^160^ ^]^

In the urinary and reproductive system, the scRNA‐seq atlas of the murine bladder described novel urothelial cells and basal cells.^[^ ^161^ ^]^ In male mice, the prostate cell atlas identified a rare luminal population with stem‐like gene expression patterns and regenerative potential.^[^ ^162^ ^]^ The murine spermatogenesis atlas identified candidate transcriptional regulators from spermatogonia to spermatids.^[^ ^163^ ^]^ By comparison, the murine testicular aging atlas observed a disrupted balance of undifferentiated and differentiated spermatogonial stem cells.^[^ ^164^ ^]^ An aging‐specific macrophage subset was also determined to contribute to a hostile proinflammatory microenvironment. In female mice, a single‐cell RNA survey of ovary development revealed germ cells and granulosa cells during primordial follicle assembly.^[^ ^165^ ^]^ Additionally, the cellular hierarchy of uterine epithelial development was also delineated.^[^ ^166^ ^]^

In the motor system, scRNA‐seq of Sox9‐positive murine skeletal progenitors predicted a bifurcated lineage, with cells differentiating toward osteogenic and adipogenic lineages. The Ccl9 signaling pathway was identified as a potential factor directing osteogenesis in bone regeneration.^[^ ^167^ ^]^ Moreover, the integration of mouse skeletal muscle single‐cell transcriptomic data resulted in a densely sampled transcriptomic model to study myogenesis, myofiber maturation, and other rare stem cell transitional states.^[^ ^168^ ^]^ Another scATAC‐seq study profiled the differentiation trajectory of murine muscle satellite cells during in vivo regeneration.^[^ ^169^ ^]^ Betaglycan was identified as a marker for the purification of muscle satellite cells. Additionally, the mouse white adipose cell atlas indicated notable cell type composition changes and higher weight gain in males with high‐fat diet‐induced obesity than in normal individuals.^[^ ^170^ ^]^

## 5 Single‐Cell Atlases of Other Organisms

### 5.1 Overview of Monkey Cell Atlas

The nonhuman primate *Macaca fascicularis* (cynomolgus) represents the closest‐to‐human alternative animal model to study human physiology, disease, and especially organ aging.^[^ ^171^ ^]^ The first nonhuman primate cell atlas (NHPCA) generated by BGI in China provided a database with more than 1 million cells.^[^ ^172^ ^]^ The cross‐tissue cell–cell interaction networks mapped the distribution of receptors and coreceptors for viruses causing human infectious diseases; the cross‐species ortholog analysis also raised potential clinical associations with human genetic disease. In the same year, another group presented a multi‐omics (RNA and ATAC) cell atlas of monkeys.^[^ ^173^ ^]^ Cross‐species comparative analyses among mice, monkeys, and humans suggested a higher degree of immune‐associated cellular similarities between monkeys and humans. In the monkey central nervous system, several studies have covered the molecular cell atlas of the retina and hippocampus.^[^ ^174^ ^]^ Furthermore, the aging cell atlas of the primate hippocampus has revealed proinflammatory responses in aged microglia and oligodendrocytes in a hostile microenvironment for neurogenesis.^[^ ^175^ ^]^ More importantly, a high‐resolution spatial transcriptomic atlas of the single‐nucleus transcriptomic landscape of primate hippocampal aging was constructed by BGI in China.^[^ ^41^ ^]^ The most comprehensive cell‐type taxonomy of the entire macaque cortex has provided insights into the cortical layer and region preferences of neural and nonneuronal cells. Notably, cross‐species comparison of transcriptomic data from humans, macaques, and mice indicated primate‐specific cell types enriched in layer 4.

Zhai and colleagues contributed to our understanding of primate development with their report on the gastrulation and early organogenesis cell atlas of monkeys.^[^ ^176^ ^]^ They demonstrated the cell‐type transcriptomic features in primitive streak development, somitogenesis, gut tube formation, neural tube patterning, and neural crest differentiation. Through cross‐species analysis, conserved Hippo signaling was identified during mesoderm differentiation among monkeys, mice, and humans. Liu's group has made significant constructions by presenting a series of primate tissue aging cell atlases in recent years. They characterized a single‐cell transcriptome atlas of aging primate cardiopulmonary,^[^ ^177^ ^]^ ovary,^[^ ^178^ ^]^ liver,^[^ ^179^ ^]^ testis,^[^ ^180^ ^]^ muscle,^[^ ^181^ ^]^ arteries,^[^ ^182^ ^]^ and pancreas.^[^ ^183^ ^]^ Most cell type‐specific aging‐associated transcriptional changes revealed increased systemic inflammation as a hallmark in primate aging. In addition, they developed tissue‐specific treatment approaches to intervene in aging‐related degeneration. All these resources have been consolidated into the Aging Atlas (https://ngdc.cncb.ac.cn/aging/index).

### 5.2 Overview of Zebrafish and Amphibian Cell Atlas

Zebrafish is an ideal model animal to study vertebrate embryogenesis. Single‐cell transcriptome analysis of zebrafish embryogenesis and development has reconstructed the transcriptional trajectories of multiple cell lineages.^[^ ^184^ ^]^ In addition, Jiang et al. represented a cross‐tissue zebrafish cell landscape with more than 250 000 single cells covering both embryo and adult stages.^[^ ^185^ ^]^ They revealed a unique characteristic of the blastema population involved in zebrafish caudal fin regeneration. Xenopus is another widely used model animal in vertebrate embryogenesis studies. Briggs et al. derived a detailed cell atlas in Xenopus and zebrafish development to identify embryonic cell states.^[^ ^186^ ^]^ This work revealed conserved and divergent features of gene expression programs in vertebrate early developmental stages. Liao and colleagues established the single‐cell transcriptome atlas of Xenopus, including multiple larval and adult organs.^[^ ^187^ ^]^ They revealed a common regulatory mechanism, including antigen processing and presentation phenotype, in cells originating from different embryo layers during metamorphosis. Axolotl is another model organism in the field of development and regeneration biology. Representative scRNA‐seq studies of axolotl limb regeneration revealed stem cell subsets and key regulatory events in fibroblasts associated with regeneration initiation.^[^ ^188^ ^]^ In addition, the first axolotl cell atlas mapped transcriptome changes during metamorphosis and development.^[^ ^189^ ^]^ Most recently, a spatial transcriptomic dataset discovered an injury‐induced ependymal cell cluster as a progenitor cell population capable of replenishing lost neurons.^[^ ^190^ ^]^ They further identified an immature neuron‐like state at the lesion site.

### 5.3 Overview of Livestock Cell Atlas

Understanding the cell‐type heterogeneity of different livestock will help the development of animal husbandry. In chicken embryos, the single‐cell transcriptome provides insights into the gonadal sex differentiation process and revealed that embryonic‐supporting cells were derived from a mesenchymal cell population.^[^ ^191^ ^]^ For pigs, two multi‐tissue single‐cell transcriptome atlases dissected the cell type‐specific regulation network and thousands of genetic variants with cell‐type interaction effects on gene expression (ieQTL).^[^ ^192^ ^]^ The latter was associated with cellular mechanisms and orthologous genes with cell‐type‐specific regulation in pigs.^[^ ^193^ ^]^ In cattle, the cross‐tissue single‐cell transcriptome landscape profiled gene expression and metabolic features of different epithelial cells related to nutrient transport.^[^ ^194^ ^]^ scRNA‐seq of mesenchymal stromal cells (MSCs) derived from horse tissue revealed that cellular motility and immune regulatory function lay the foundation for the therapeutic potential of MSCs.^[^ ^195^ ^]^ In addition, some tissue cell atlases have been constructed in sheep, snake, and deer. For example, a scRNA‐seq study of sheep spermatogenesis found that the ribosome pathway was significantly enriched in testicular somatic cell types.^[^ ^196^ ^]^ A scRNA‐seq survey of snake venom gland organoids identified distinct venom‐expressing cell types and proliferative cells with homologs of known mammalian stem cell markers.^[^ ^197^ ^]^ scRNA‐seq of antler regrowth identified a population of “antler blastema progenitor cells” derived from mesenchymal cells with regeneration ability.^[^ ^198^ ^]^

Moreover, two systematic single‐cell atlases of multiple nonmodel mammals, reptiles, and birds provided more valuable data for deeper cross‐species analysis in evolution and infection studies.^[^ ^199^ ^]^ Notably, rabbits have emerged as a promising model for single‐cell comparative genomics. The single‐cell landscape of rabbit gastrulation and early organogenesis has provided an in‐depth resource for cross‐species studies with other animals. Gene regulatory programs between rabbits and mice identified regions of similarity and diversity across species.^[^ ^200^ ^]^

### 5.4 Overview of Fly and Worm Cell Atlas

The fruit fly (*Drosophila melanogaster*) and *Caenorhabditis elegans* are both classical model organisms in genetics. Li and colleagues have reported cross‐tissue single‐cell transcriptome atlas of adult and aging flies. The adult fly cell atlas included gene signatures and transcription factors related to common cell types.^[^ ^201^ ^]^ The cellular signatures for each tissue will serve as a reference for genetic perturbation studies and disease models. The aging fly cell atlas provided insights into age‐related gene expression and pathway changes,^[^ ^202^ ^]^ such as the observed lineage shift to the fat body and apoptosis in muscles. Additionally, an aging clock model was established, allowing the prediction of an animal's age using scRNA‐seq data. Regarding the fly brain, two other research teams have deciphered the single‐cell accessible chromatin and transcriptome atlas of adulthood and aging. Comprehensive regulatory regions in multiple neural cell types were determined to be associated with developmental trajectories involving neurogenesis, reprogramming, and maturation.^[^ ^203^ ^]^ Notably, the atlas of the aging fly brain revealed exponential declines in RNA content and regulatory states related to oxidative phosphorylation.^[^ ^204^ ^]^

To remove tissue batch effects, the whole‐body cell landscapes of zebrafish, flies, and earthworms have been constructed.^[^ ^205^ ^]^ Among the cell types in the whole‐body fly cell atlas, ≈93.1% were consistent with those in the cross‐tissue fly cell atlas.^[^ ^201^ ^]^ The earthworm cell atlas provides more major cell lineages than previously studied, not just muscle‐related cells.^[^ ^206^ ^]^ The developmental *C. elegans* cell atlas covers whole‐body cells in a single organism. Thus, the lineage‐resolved molecular atlas of *C. elegans* embryogenesis and larval development revealed lineage priming of specific cells until terminal fate decision.^[^ ^207^ ^]^ Other single‐cell transcriptome atlases of mosquitos and malaria (*Plasmodium berghei*) both focused on human pathogen transition.^[^ ^208^ ^]^ The hemocyte lineages in mosquitoes have identified the cellular events that underpin mosquito immunity to malaria infection.

### 5.5 Overview of Marine Organism Cell Atlas

Marine life contains great treasures to help the development of evolution, natural product synthesis, and ecology. In 2018, two crucial single‐cell transcriptome atlases of planarian *Schmidtea mediterranea* were constructed, shedding light on the cell types with lineage progenitors for differentiated cells, especially pluripotent stem cells.^[^ ^209^ ^]^ Coregulated gene sets during the differentiation of many specific cell types in regenerating planarians were evaluated. In another important work by Zeng et al., Tspan‐1‐positive neoblasts were successfully identified in adult planaria as pluripotent stem cells. These cells survived sublethal irradiation and underwent a clonal expansion to repopulate whole animals, as validated using a single‐cell transplantation experiment of lethally irradiated animals.^[^ ^210^ ^]^ Taking advantage of the spatial transcriptome, Cui et al. reported a 4D spatiotemporal transcriptomic cell atlas of the planarian regeneration process.^[^ ^211^ ^]^ They also provided a public online spatiotemporal analysis database for planarian regeneration research.

**Figure 3:**
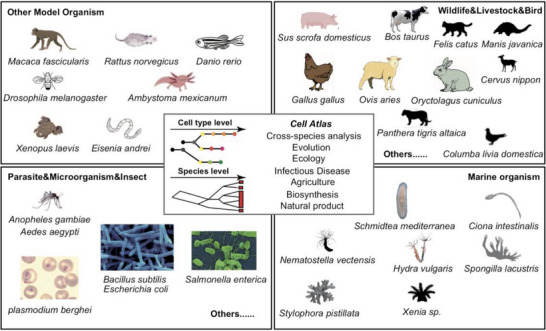
Current advances of organism cell atlas. Not including the cell atlas of plants. Researchers have generated rich single‐cell atlas resources of the most widely studied model organisms to help the development of genetics and basic biology science. To push forward the cross‐species study, the current reported cell atlas may include but are not limited to wide‐animal, birds, livestock, parasites, microorganism, insects, and marine organisms. Single‐cell atlas of multiple organisms enabled further development in evolution in single‐cell resolution.

In 2018, using whole‐organism scRNA‐seq of cnidarians (cnidarian *Nematostella vectensis*), regulator enrichment analysis of cell lineages revealed a lineage‐specific diversification event in neurons.^[^ ^212^ ^]^ In 2019, the single‐cell transcriptome atlas of Hydra identified molecular signatures in the cell renewal process. Differentiation trajectories from stem cells to terminally differentiated cells enriched putative regulators and gene modules of the self‐renewing and nerve systems.^[^ ^213^ ^]^ In the same year, a single‐cell transcriptome analysis of a proto‐vertebrate (*Ciona intestinalis*) was used to construct virtual cell‐lineage maps and provisional gene networks for 41 neural subtypes.^[^ ^214^ ^]^ Based on transcriptome trajectory analysis, a model was built for the evolution of the telencephalon. To study the evolutionary origin of neurons and muscles, Musser et al. performed whole‐body scRNA‐seq of sponges (*Spongilla lacustris*), an animal without a nervous system or musculature.^[^ ^215^ ^]^ The specialized secretory neuroid cells were morphologically validated with secretory vesicles and cellular projections enwrapping choanocyte microvilli and cilia, which is the conserved prototype of pre‐ and postsynapses in the nervous system. In addition, an organism‐wide, single‐cell transcriptomic atlas of the hydrozoan medusa *Clytia hemisphaerica* revealed a cell state shift under starvation conditions.^[^ ^216^ ^]^ Moreover, a genome assembly and scRNA‐seq atlas of *Xenia species* (coral) identified the endosymbiotic cell type that contributed to the immune modulation of host coral cells.^[^ ^217^ ^]^ For the stony coral (*Stylophora pistillata*), a single‐cell transcriptome atlas has been constructed to discover specialized immune cells and dynamic gene expression patterns of calcium‐carbonate skeleton formation.^[^ ^218^ ^]^ Cross‐species data integration of multiple cnidarian cell type atlases revealed the coral cell specialization process (**Figure** **3**).

## 6 Single‐Cell Atlases in Disease Biology

**Figure 4:**
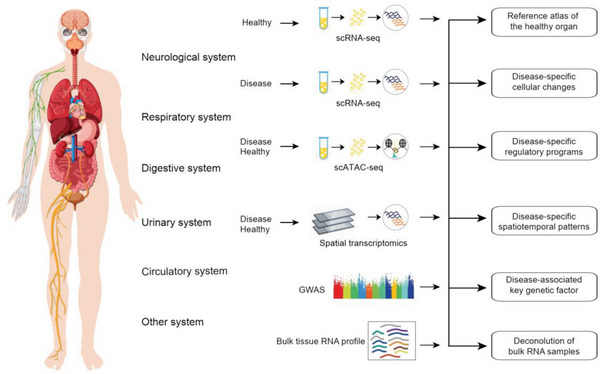
Overview of study design and analysis pipeline of single cell atlas in disease biology. These ongoing cell atlases can be used for i) reference atlas of healthy data, ii) disease‐specific cell types, iii) disease‐specific regulatory programs, iv) disease‐specific spationtemporal patterns, v) disease‐specific key genetic mutations, and vi) bulk RNA disease samples deconvolution on single cell atlas.

The occurrence of disease is a highly complex process arising from aberrations in cells and cellular ecosystems within tissues. The advent of state‐of‐the‐art, large‐scale, and single‐cell omics technologies has revolutionized our ability to explore the cellular landscape of disease at an unprecedented level. These advanced techniques go beyond mere descriptive features, allowing us to delve deeper into disease mechanisms and guide functional studies. Through the integration of multiple omics datasets, single‐cell atlases provide innovative insights, bridging the gap between genetic biology and disease biology.^[^ ^62^ ^]^ Currently, single‐cell atlases in disease biology have been established for eight major systems within the human body (**Figure** **4**).

### 6.1 Single‐Cell Atlases in Urinary Disease

Acute kidney injury (AKI) and Chronic kidney disease (CKD) are the two most devastating categories of urinary disease that occur after kidney injury. To better understand kidney disease, researchers have focused on defining the complexity of cell types, states, programs, and contexts in which disease‐implicated genes act. Integrative analyses of multi‐omics single‐cell data from healthy and diseased kidneys have been achieved, which revealed novel unexpected associations. For example, by integrating multiomics data from healthy kidney cell types and constructing a reference atlas at a single‐cell resolution,^[^ ^92^, ^94^ ^]^ researchers have identified major cell types and subcell types in the kidney, providing a detailed landscape of the cellular and molecular composition of the kidney. Integrated healthy kidney atlases have revealed the presence of a subpopulation of proximal tubules that expresses *VCAM1*, called PT_VCAM1, which is identified and recognized as an injured cell state.^[^ ^94^ ^]^ Through the further integration of healthy and disease atlases, 28 cellular states across nephron segments and the interstitium were determined to be altered in kidney injury.^[^ ^219^ ^]^ Moreover, the spatial mapping of injury neighborhoods shows that altered cellular states are enriched in the proximal tubule and thick ascending limb (TAL) neighborhoods, with distinct immune‐active cellular niches associated with healthy and injured tubules.^[^ ^219^ ^]^ By merging the extensive GWAS data with the comprehensive integrated cell atlas, researchers have facilitated the identification of key genetic factors and regulatory regions that contribute to kidney disease development. Notably, the altered TAL regulatory regions were linked to estimated glomerular filtration rate (eGFR) and chronic kidney failure, and estrogen‐related receptor motifs are also implicated in this context.^[^ ^219^ ^]^

### 6.2 Single‐Cell Atlases in Respiratory Disease

Lung disease is the primary cause of death worldwide. As pulmonary diseases are marked by the emergence of unique disease‐associated transcriptional phenotypes, the construction of a reference atlas of the healthy human lung^[^ ^72^ ^]^ could be instrumental in facilitating mapping the changes in the cellular landscape during lung disease. Focusing mainly on a specific disease, the integration of transcriptome data from both healthy and diseased patients has yielded valuable insights into specific molecular changes occurring in lung disease, such as altered epithelial cells of the airway wall during asthma pathogenesis^[^ ^220^ ^]^ as well as abnormal diversity of endothelium and stroma cells of the distal lung parenchyma in idiopathic pulmonary fibrosis (IPF).^[^ ^221^ ^]^ To construct a universal reference, a recent study consolidated an integrated human lung cell atlas (HLCA)^[^ ^74^ ^]^ combining 49 datasets of the human respiratory system into a single atlas. Mapping single cells of diseased individuals to the HLCA, more uncertainty and altered cell states were revealed in lung disease; deconvolution of bulk RNA samples from lung disease using the HLCA, disease‐specific changes in cell type composition were revealed; mapping associated results from GWAS to HLCA, crucial links between genetic predispositions and cell types were established to provide essential context for understanding disease.

### 6.3 Single‐Cell Atlases in Digestive Disease

Gut diseases are common complex digestive disorders. Foundational studies have begun to map the cellular landscape of a healthy human intestine, cataloging cell types throughout the intestine^[^ ^82b^ ^]^ and further extending the organization of cell types in the intestines by spatial distribution as well as epigenetic regulatory information.^[^ ^84^ ^]^ As mentioned above, the integrated healthy gut cell atlas can be used to reveal meaningful differences between healthy and diseased tissue, such as contextualizing GWASs. Notably, inflammatory bowel disease (IBD) has gained widespread attention, with 12 IBD‐related studies published over the last 5 years. These studies involved 440 samples, revealing distinct cell lineages that contribute to IBD. Using these datasets, the scIBD platform^[^ ^222^ ^]^ has been constructed to integrate large‐scale datasets and further systematic investigation of IBD. The scIBD framework facilitates comparative studies of different single‐cell datasets in a holistic manner, encompassing various aspects, such as the investigating intestinal microenvironment, exploring disease‐associated immune and nonimmune cell subsets, unraveling the cell‐type specificity of GWAS‐risk genes, and examining the cell‐type specificity of drug targets for IBD.

### 6.4 Single‐Cell Atlases in Circulatory System Disease

Cardiovascular disease is the leading cause of death worldwide. The cell atlas facilitates the exploration of cardiovascular disease at an unprecedented level.^[^ ^223^ ^]^ To achieve advanced insights into disease mechanisms and develop effective therapeutic strategies, integrated analyses have been conducted on healthy hearts to identify specific cell types.^[^ ^79^ ^]^ Furthermore, the integration of spatial data has allowed for the identification of specific cardiac niches^[^ ^80^ ^]^ and a better understanding of the spatiotemporal patterns in human cardiogenesis.^[^ ^81^ ^]^ Integrated transcriptomic landscapes have been constructed for specific heart diseases, such as heart failure,^[^ ^224^ ^]^ hypertrophic cardiomyopathy,^[^ ^225^ ^]^ and congenital heart block (CHB).^[^ ^226^ ^]^ By integrating data obtained from multi‐omic techniques, integrated cell atlases of congenital heart disease (CHD)^[^ ^227^ ^]^ and myocardial infarction^[^ ^228^ ^]^ have been constructed for more comprehensive studies on these conditions. In the context of atherosclerosis, recent integrated transcriptomic cell atlases have yielded valuable cellular insights, such as immune^[^ ^229^ ^]^ and nonimmune^[^ ^230^ ^]^ cell composition of atherosclerotic plaques,^[^ ^230^ ^]^ and the transitional state in late‐stage atherosclerosis.^[^ ^231^ ^]^ Additionally, an integrated atlas of chromatin accessibility in human coronary arteries has highlighted regulatory mechanisms associated with the risk of coronary artery disease (CAD).^[^ ^232^ ^]^

### 6.5 Single‐Cell Atlas in Neurological Disorders

Alzheimer's disease (AD) and Parkinson's disease (PD) are two of the most common neurodegenerative diseases globally. In the context of AD, a cell atlas was initially constructed from transcriptional data.^[^ ^233^ ^]^ This was followed by the integration of epigenetic regulation^[^ ^234^ ^]^ and then the integration of spatial data to construct a more comprehensive integrated cell atlas.^[^ ^235^ ^]^ In the context of PD, cell atlases have been constructed based on transcriptional data^[^ ^236^ ^]^ and the integration of transcriptomic and spatial transcriptomics data.^[^ ^237^ ^]^ They highlight “panglial” activation as a central mechanism in the pathology of the movement disorder^[^ ^236a^ ^]^ and identify a specific subpopulation, SOX6_AGTR1, which exhibits high susceptibility to neurodegeneration in PD.^[^ ^237^ ^]^

### 6.6 Single‐Cell Atlases in Other Diseases

Although muscle, bone, cartilage, and other musculoskeletal tissues pose challenges in terms of digestion, the utilization of single‐cell and spatial omics in musculoskeletal disorder research^[^ ^238^ ^]^ has greatly enhanced our understanding of these conditions. Recently, MSdb^[^ ^239^ ^]^ collected 121 samples from individuals with musculoskeletal disorders, including degenerative spine disease, arthritis, osteoarthritis, and osteosarcoma. An integrated cell atlas of transcriptomic and spatial transcriptomic analyses in skeletal muscle fibrosis^[^ ^240^ ^]^ has shown that gal‐3+ macrophages are activated in response to acute injury. In the context of secretory system disease, researchers have integrated chromatin accessibility and gene expression at the single‐cell level to address sample size limitations and confounding factors.^[^ ^241^ ^]^ This study utilized human islet preparations from 34 donors, including nondiabetic, type 2 diabetes (T2D), and pre‐T2D donors, to define regulatory programs involved in T2D pathogenesis.^[^ ^242^ ^]^ Recent research efforts have focused on constructing comprehensive cellular atlases of the human reproductive system throughout various stages of life.^[^ ^243^ ^]^ These atlases have proven invaluable in enhancing our understanding of reproductive pathologies such as infertility, endometriosis, and ovarian cancer.

## 7 Deep Learning Application in a Single‐Cell Atlas

Deep learning (DL) is a powerful method for modeling complex relationships within high‐dimensional data and helps to learn mappings from genome sequences to gene expression under certain conditions. The categorization of DL models can be based on whether the input feature is a DNA sequence, leading to the classification of models into sequence‐based and nonsequence‐based models.

Currently, nonsequence‐based models are capable of holistically accounting for all stages of scRNA‐seq analysis,^[^ ^244^ ^]^ including normalization such as scVI,^[^ ^245^ ^]^ data correction such as ResNet^[^ ^246^ ^]^ and DESC,^[^ ^247^ ^]^ clustering and cell annotation such as scVAE^[^ ^248^ ^]^ and scDFC,^[^ ^249^ ^]^ cell–cell communication analysis,^[^ ^250^ ^]^ and RNA velocity such as DeepVelo.^[^ ^251^ ^]^ Notably, Geneformer,^[^ ^252^ ^]^ the first large model of computational biology, was pretrained on 30 million single‐cell transcriptomes to achieve predictions using transfer learning. In addition, with the advancement of spatial data, an increasing number of DL models are being designed specifically for spatial data. To capture both gene expression profiles and spatial information, the models DeepST,^[^ ^253^ ^]^ SpaCell,^[^ ^254^ ^]^ GraphST,^[^ ^255^ ^]^ STAGATE,^[^ ^256^ ^]^ and DeLTA2.0^[^ ^257^ ^]^ were developed. To identify continuums of cell types in spatial data, DestVI^[^ ^258^ ^]^ utilizes a reference of the scRNA‐seq dataset to deconvolve within a given spatial transcriptomics (ST) spot. To infer cell‒cell interactions (CCIs) at a single‐cell spatial resolution, graph convolutional networks (GCNs), such as GCNG^[^ ^259^ ^]^ and HoloNet,^[^ ^260^ ^]^ are commonly employed. In the integration of multimodal omics data, the main advantage of GCNs lies in their ability to handle data with incomplete spatial relationships and leverage the power of convolutional networks.^[^ ^261^ ^]^ Examples include GCN‐SC,^[^ ^262^ ^]^ graph‐based autoencoder (AE) models,^[^ ^263^ ^]^ and variational graph autoencoders (VGAEs) such as GLUE.^[^ ^264^ ^]^

The significance of sequence‐based DL models lies in their ability to reliably predict gene expression directly from sequences. They elucidate how a single genome can encode distinct gene expression profiles in different cell types. Sequence‐based DL models have increasingly been used for analyzing and predicting various molecular features in single‐cell data, including chromatin accessibility and gene expression.^[^ ^265^ ^]^ For example, Basset^[^ ^266^ ^]^ and DeepFlyBrian^[^ ^203^ ^]^ have been employed to predict chromatin accessibility from DNA sequence information at the pseudobulk level.^[^ ^25^, ^203^ ^]^ Similarly, other deep learning models have been developed for analyzing scATAC‐seq data, which provides information about chromatin accessibility at single‐cell resolution. This includes models such as scBasset,^[^ ^267^ ^]^ which achieves state‐of‐the‐art performance across a variety of tasks on scATAC‐seq data, including cell clustering, data denoising, data integration across assays, and transcription factor activity inference. Notably, Nvwa^[^ ^205^ ^]^ is a groundbreaking model that revolutionizes gene expression at the single‐cell level across different species. Inspired by the ancient Chinese legend of Nvwa, a powerful mother god who possesses transformative abilities, the Nvwa model aims to map DNA sequences at the single‐cell level by creating a unified framework of the regulatory process of various gene expressions and analyzing how different cell types use the same DNA sequence to encode different genes. Initially, independent Nvwa models were trained on eight species to evaluate their accuracy in predicting single‐cell gene expression. Remarkably, the Nvwa model successfully predicted genome‐wide transcriptional activity signals and demonstrated a correlation with experimental functional genomics data. To understand the mechanisms behind the accurate predictions of the Nvwa models, the researchers examined the sequence patterns learned by the models. Nvwa effectively utilized DL‐derived *cis*‐regulatory elements (CREs) with specific cell types and identified complex regulatory rules. These findings shed light on the conservation and divergence of DL‐based CREs across species, providing insights into the cross‐species genetic network. Furthermore, Nvwa systematically compared cell type‐specific transcription factors to uncover conserved genetic regulations in both vertebrates and invertebrates. This work establishes Nvwa as a valuable resource and presents a novel strategy for studying regulatory grammar in diverse biological systems.

In addition, sequence‐based DL models can be used to predict the regulatory effect of DNA variants by comparing the epigenomic properties of different alleles.^[^ ^268^ ^]^ Some examples of this type of model are DeepSea,^[^ ^269^ ^]^ ExPecto,^[^ ^270^ ^]^ Enformer,^[^ ^271^ ^]^ and Basset,^[^ ^266^ ^]^ which can provide single‐nucleotide and allele‐specific predictions of variant effects on epigenomic features in bulk samples. Additionally, scBasset^[^ ^267^ ^]^ can perform in silico saturation mutagenesis (ISM) on a 100‐bp sequence. scBasset allows for the prediction of changes in accessibility for every cell after mutating each position to its three alternative nucleotides. Furthermore, MetaChrom^[^ ^272^ ^]^ is a model that utilizes transfer learning to predict neurodevelopment‐specific variant effects. By integrating GWAS results, MetaChrom has identified 31 likely functional SNPs in 30 genetics studies of schizophrenia (SCZ)‐associated loci. To map genetic variants to the cell‐type level, a state‐of‐the‐art model called Huatuo^[^ ^273^ ^]^ has been developed. Huatuo is a framework that aims to decode the genetic variation of gene regulation at both cell‐type and single‐nucleotide resolutions. With the advantages of HCL, significant progress has been made in the Huatuo workflow. One notable achievement is the improved DL model, which effectively analyzes and interprets the effect of mutations on gene expression in different cell types. In addition, a novel approach has been proposed to infer cell type‐dependent expression quantitative trait loci (eQTLs). This innovative approach leverages HCL to unravel the intricate relationships between genetic variations and gene expression patterns specific to different cell types. By incorporating cell type‐specific information, researchers can gain a deeper understanding of how genetic variations influence gene expression in a context‐dependent manner. This framework facilitates the investigation of cell landscapes and genome‐wide genetic variations in cell type‐specific regulation using scRNA‐seq data from a small cohort of individuals. Huatuo takes inspiration from the renowned ancient Chinese physician Hua Tuo, who is considered one of the most famous physicians in ancient China. By adopting the Huatuo workflow, researchers can acquire valuable insights into complex traits and diseases, including potential driver cell types and functional mechanisms of trait‐causal and disease‐causal genetic variations. Notably, other frameworks also leverage pretrained epigenome models to predict single‐cell gene expression, such as ExpectoSC^[^ ^274^ ^]^ and seq2 cells.^[^ ^275^ ^]^

## 8 Conclusion and Future Perspectives

Single‐cell atlases have greatly promoted the development of integrative biology with far‐reaching implications for various fields. They have deepened our knowledge of fundamental biological processes and mechanisms, shedding light on previously unknown cell types and their roles in development, homeostasis, and disease. This understanding has paved the way for new avenues of investigation and discovery. In clinical practice, cell atlases offer the potential for improved diagnostics, prognostics, and personalized medicine. By comparing healthy and diseased tissues at the single‐cell level, researchers can identify specific cell types or states associated with diseases, aiding in early detection and targeted interventions.

The application of machine learning techniques in single‐cell atlases promotes the development of systems biology to predictive biology. Artificial intelligence (AI) algorithms can uncover intricate patterns within the vast amount of data generated by cell atlases, enabling the prediction of gene expression and the reconstruction of regulatory networks. This data‐driven approach complements traditional biological insights, accelerating discoveries and facilitating the identification of novel biomarkers and therapeutic targets.

**Figure 5:**
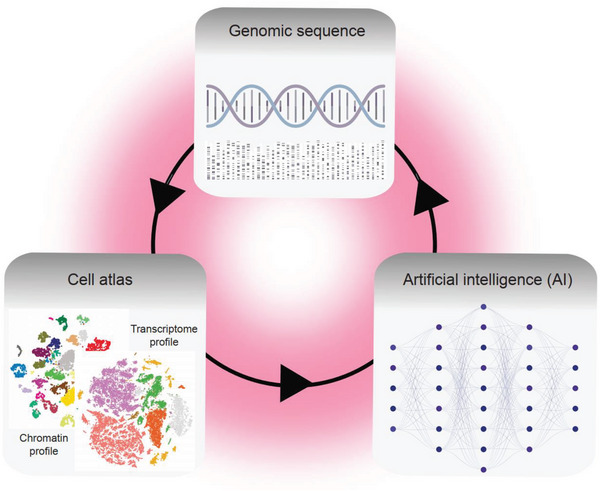
Future experimental strategy. Integrating genomics, single‐cell atlases, and AI to play pivotal roles in shaping the future of predictive biology.

In the future, the synergy among single‐cell omics technology, cell atlases, and AI has great potential for advancing genomics research. The future of genomics research is shaped by the integration of state‐of‐the‐art AI models with single‐cell atlas data (**Figure** **5**), enabling us to predict and understand various aspects of life at an unprecedented level.

## Conflict of Interest

The authors declare no conflict of interest.
